# Data on antiplasmodial and stage-specific inhibitory effects of Aromatic (Ar)-Turmerone against *Plasmodium falciparum* 3D7

**DOI:** 10.1016/j.dib.2020.106592

**Published:** 2020-11-26

**Authors:** Amatul Hamizah Ali, Hani Kartini Agustar, Nurul Izzaty Hassan, Jalifah Latip, Noor Embi, Hasidah Mohd Sidek

**Affiliations:** a Department of Chemical Sciences, Faculty of Science and Technology, Universiti Kebangsaan Malaysia, 43600 UKM Bangi, Selangor, Malaysia; b Department of Earth Sciences and Environment, Faculty of Science and Technology, Universiti Kebangsaan Malaysia, 43600 UKM Bangi, Selangor, Malaysia; c Department of Biological Sciences and Biotechnology, Faculty of Science and Technology, Universiti Kebangsaan Malaysia, 43600 UKM Bangi, Selangor, Malaysia

**Keywords:** *Curcuma longa*, Aromatic-turmerone, Antiplasmodial activities, *Plasmodium falciparum*, Stage-specific inhibition

## Abstract

Aromatic (ar)-turmerone is one of the aromatic constituents abundant in turmeric essential oil from *Curcuma longa*. Ar-turmerone exhibited anti-inflammatory properties. So far, antiplasmodial data for ar-turmerone is still not reported. The data showed the *in vitro* antiplasmodial effect of ar-turmerone against *Plasmodium falciparum* 3D7 (chloroquine-sensitive) via Plasmodium lactate dehydrogenase assay (pLDH) and cytotoxic effect against Vero mammalian kidney cells using 3-(4, 5-dimethylthiazol-2-yl)-2, 5-diphenyltetrazolium bromide (MTT) colourimetric assay. Selectivity indexes of ar-turmerone were calculated based on inhibition concentration at 50% of parasite growth (IC_50_) from MTT and pLDH assays and the effects of ar-turmerone were compared to the antimalarial reference drug chloroquine diphosphate. The inhibitory effect of ar-turmerone at the intraerythrocytic stages of plasmodial lifecycles was evaluated via a stage-dependant susceptibility test. The antiplasmodial and cytotoxic activities of ar-turmerone revealed IC_50_ values of 46.8 ± 2.4 μM and 820.4 ± 1.5 μM respectively. The selectivity index of ar-turmerone was 17.5. Ar-turmerone suppressed the ring-trophozoite transition stage of the intraerythrocytic life cycle of *P. falciparum* 3D7.

**Specifications Table**

**Table:** 

Subject	Biology
Specific subject area	Developmental biology
Type of data	Table and Figure
How data were acquired	- Antiplasmodial test; Plasmodium lactate dehydrogenase (pLDH) colorimetric test - Cytotoxic test; 3-(4, 5-dimethylthiazol-2-yl)-2, 5-diphenyltetrazolium bromide (MTT) colorimetric test - Stage-specific susceptibility test; light microscopy examination
Data format	Raw
Parameters for data collection	IC_50_ calculated from antiplasmodial and cytotoxic tests were used to measure the selectivity index of ar-turmerone. SI value was measured from the ratio of IC_50_ of cytotoxicity over IC_50_ of antiplasmodial activity. Chemo-suppression was measured by counting the number of new rings formed during the intraerythrocytic cycle for test compound as compared to control. Next, changes with the morphology of the parasite after ar-turmerone treatment were monitored using thin and thick blood smear techniques.
Description of data collection	The effects of antiplasmodial and cytotoxic of ar-turmerone were evaluated on *Plasmodium falciparum* 3D7 (chloroquine-sensitive) and Vero mammalian kidney cells respectively. Stage-specific inhibition of ar-turmerone was monitored at ring, trophozoite, and schizont of intraerythrocytic stages of *P. falciparum* lifecycles.
Data source location	Department of Biological Sciences and Biotechnology, Faculty of Science and Technology, Universiti Kebangsaan Malaysia, 43600 UKM Bangi, Selangor, Malaysia.
Data accessibility	Repository name: Mendeley Data Data identification number: 10.17632/wvnb772htc.1 Direct URL to data: https://data.mendeley.com/datasets/wvnb772htc/draft?a=c9cba652-8b12-41dc-bcae-1bb10d27defe

## Value of the Data

• The research data is important to understand the *in vitro* antimalarial and cytotoxic properties of Ar-turmerone which is useful for further animal (*in vivo*) and clinical studies.
• The data obtained from this work will add to the knowledge about the medicinal value of *Curcuma longa* as an adjunctive therapy remedy for malaria treatment.
• Parasitologists, pharmacologists, pharmacists, and nutritionists can give thought to the data obtained as it may be a new antimalarial drug template and as natural supplements for immunity against malarial infection.
• The research data is useful as a reference for further drug development of experiments for malarial drug discovery involving drug pharmacology, toxicity and pharmacokinetic fields.

## 1 Data Description

**Fig. 1:**
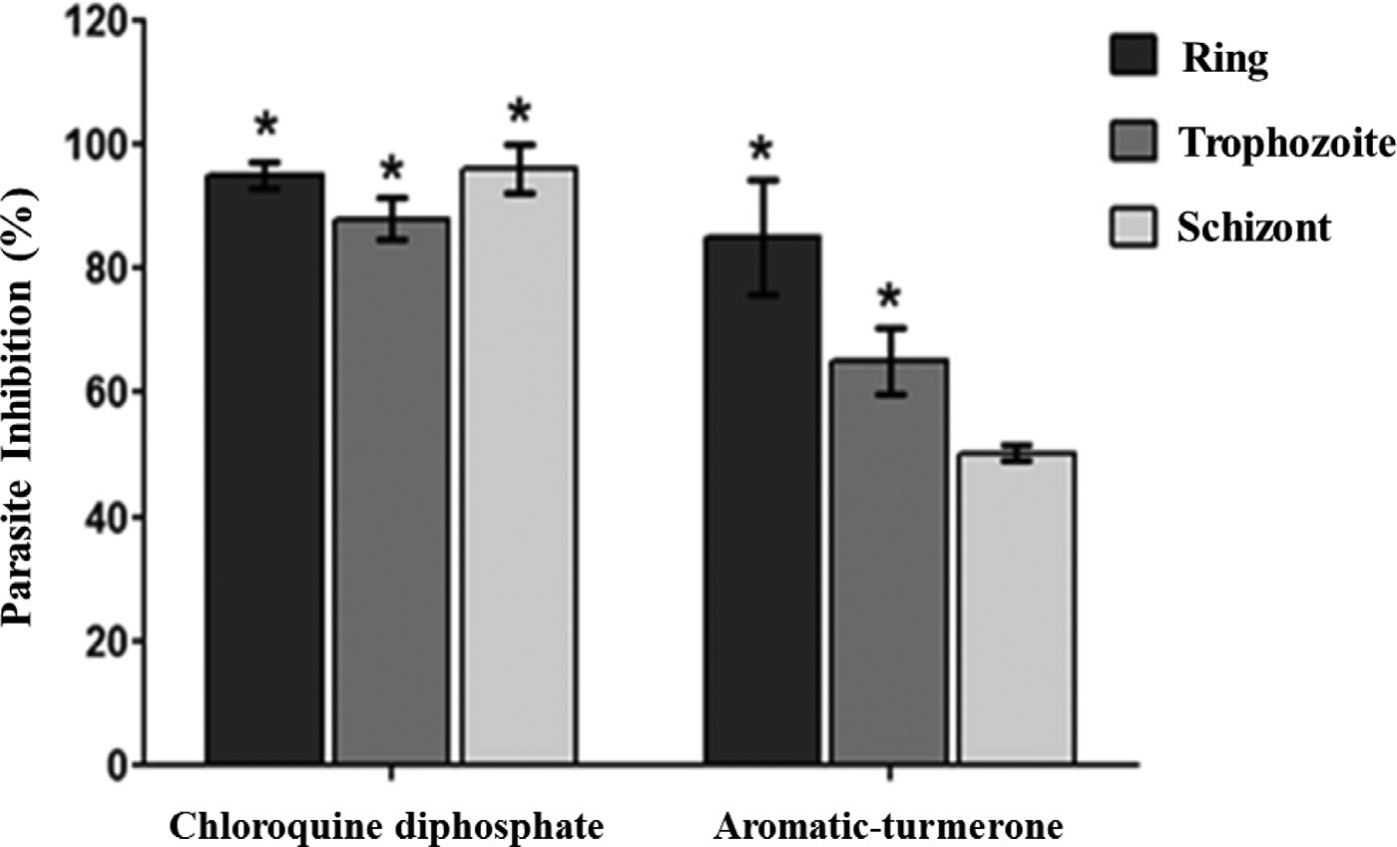
Inhibitory effect after 5 hours exposure of ar-turmerone in different intraerythrocytic stages of *P. falciparum* 3D7. Parasite inhibition was measured by the ratio of parasitaemia level in treated cultures over untreated cultures. Chloroquine diphosphate treatment represented as a reference drug. Each bar corresponded to the mean with SD from three experiments. Sign (*) indicates a significant result (p<0.05) as compared to control based on one-way ANOVA analysis.

**Fig. 2:**
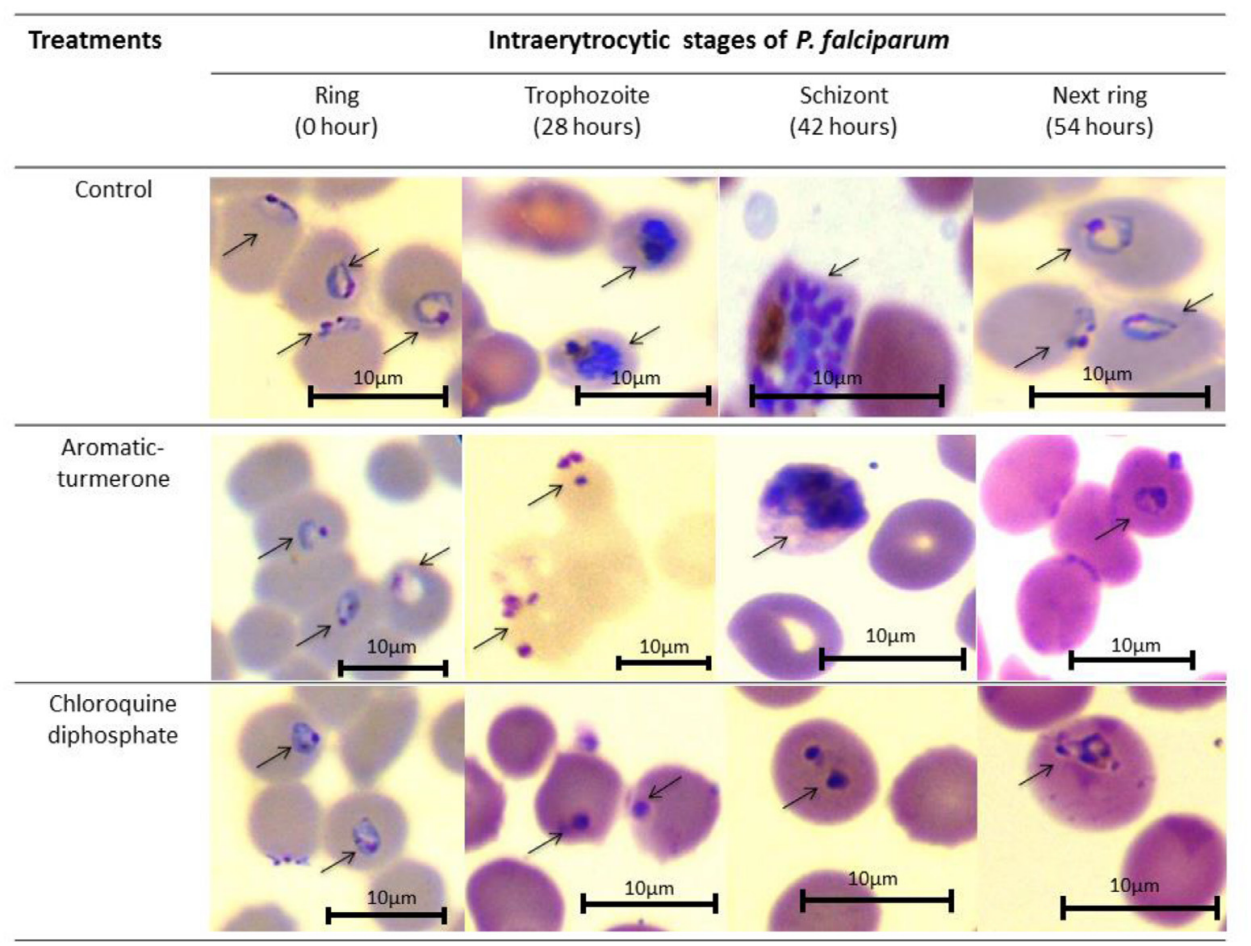
Morphological changes of parasites were observed after incubation with ar-turmerone or chloroquine diphosphate. Highly synchronous *P. falciparum* 3D7 cultures were treated with the IC_90_ value of ar-turmerone (463.5 µM) or chloroquine diphosphate (0.052 µM), determined by non-linear regression analysis from pLDH assay. Chloroquine diphosphate treatment is represented as a reference drug. Arrows show the presence of parasites in the erythrocytes. The red or purple stain is referred to as erythrocytes, while blue or dark blue stain is referred to as parasites. Colour differences between the microscopic images are due to white balance settings and this does not affect the analysis of the result.

The data on *in vitro* antiplasmodial and cytotoxic activities of ar-turmerone were reported in this dataset. The antiplasmodial effect of ar-turmerone against *P. falciparum* 3D7 (chloroquine-sensitive) using plasmodium lactate dehydrogenase (pLDH) assay showed an IC_50_ of 46.8 ± 2.4 µM as demonstrated in Table 1. The raw data source for this dataset is available through the Mendeley database link (Table S1). The data for the cytotoxic effect of ar-turmerone (IC_50_ = 820.4 ± 1.5 µM) was also shown in Table 1. Chloroquine diphosphate (pLDH IC_50_=0.0058±0.0012 µM; MTT IC_50_= 1020.9±10.7 µM) was used as an antimalarial reference drug. The selectivity index (SI) of ar-turmerone showed a value of 17.5. This data suggested that ar-turmerone is a promising antiplasmodial compound and non-toxic. Chloroquine diphosphate exhibited high SI value (SI>2000). High SI value shown by the antimalarial drug, chloroquine diphosphate indicated that the drug is potent and non-toxic. The stage-inhibitory effect of ar-turmerone revealed 85% suppression at the ring stage, 65% suppression at the trophozoite stage, and 50% suppression at the schizont stage (Fig. 1). The raw data source for this dataset is also available through the Mendeley database link (Table S2). The examination of parasite morphology changes in *P. falciparum* intraerythrocytic stages was presented in Fig. 2. Positive inhibitory effects of ar-turmerone were found at the ring-trophozoite transition stage of the parasite intraerythrocytic life cycle. The rings underwent lyses and loss of cellular architectures. For the trophozoite-schizont transition stage, a longer transition period was observed in ar-turmerone treatment showing that development of the parasite intraerythrocytic morphologies from trophozoite to schizont had been slowed down due to the effect of ar-turmerone. However, some of the parasites (>60%) still survived indicating that the treatment of ar-turmerone during the trophozoite-schizont transition stage did not effectively eradicate the parasites compared to the ring-trophozoite transition stage. Parasites from the schizont-ring transition stage treated with ar-turmerone did not show any significant morphological changes. For an antimalarial reference drug, chloroquine diphosphate, inhibition of parasite was seen at all stages in the intraerythrocytic parasite life cycle. The data source (Table S1 and Table S2) for this data is available in Mendeley database link (https://data.mendeley.com/datasets/wvnb772htc/draft?a=c9cba652-8b12-41dc-bcae-1bb10d27defe).

**Table 1:** *In vitro* antiplasmodial and cytotoxicity activities of aromatic-turmerone against *P. falciparum* 3D7.

Compound/ drugs	Antiplasmodial activity, pLDH assay, IC_50_ (µM)±SD	Cytotoxic effect, MTT assay, IC_50_ (µM) ±SD	$\text{Selectivity}\;\text{Index}\;\left(\text{SI}\right)=\left(\frac{IC_{50}\;\mathrm{MTT}}{IC_{50}\;\mathrm{pLDH}}\right)$
Aromatic-turmerone	46.8±2.4	820.4±1.5	17.5
Chloroquine diphosphate	0.0058±0.0012	1020.9±10.7	>2000

SD: standard deviation

## 2 Experimental Design, Materials and Methods

### 2.1 *In vitro* antiplasmodial assessment

Chloroquine-sensitive strain (3D7) of *P. falciparum* was collected from the Malaria Research and Reference Reagent Resource Centre (MR4), Virginia, USA. The parasite was revived from cryopreservation and cultured in growth medium at 2% haematocrit in purified O+ human erythrocytes at 37 °C, 5% CO_2_. Human erythrocytes were provided by the University Health Centre. This study protocol received ethical approval from the Research Ethics Committee of Universiti Kebangsaan Malaysia (JEP-2020-334). In this study, all chemicals and reagents were purchased from Sigma-Aldrich, St. Louis, MO, USA unless stated otherwise. The growth medium contained RPMI 1640 (Gibco, Life Technologies, USA) supplemented with 0.5% Albumax I (Gibco, Life Technologies, USA), 25 mM N-2-hydroxyethylpiperazine-N-2-ethanesulfonic acid (HEPES) (Gibco, Life Technologies, USA), 100 μM hypoxanthine, 12.5 μg/mL gentamicin and 1.77 mM sodium bicarbonate [1]. The parasitaemia level was monitored by microscopic examination using thin and thick blood smears. Susceptibility of the *P. falciparum* 3D7 to ar-turmerone (Cayman Chemicals, Ann Arbor, MI, USA) (CAS Number:532-65-0) was evaluated using parasite lactate dehydrogenase (pLDH) assay. PLDH assay was conducted in flat-bottomed 96-well microtiter plates [2]. Before the incubation started, the parasite cultures were synchronised to obtained >90% rings using 5% D-sorbitol. Parasites were later plated at 2% haematocrit and 2% parasitaemia in culture media containing ar-turmerone at concentration ranged from 0.01 to 1000 µM and incubated for 48 h. Chloroquine diphosphate (CAS Number: 50-63-5) was used as antimalarial reference drug at concentration ranged from 0.0001 to 100 µM. Malstat reagent was prepared by mixing sodium lactate (4 g), Tris buffer (1.32 g), triton X-100 (400 µL) and 3-acetylpyridine adenine dinucleotide (APAD) (0.02 g) in 200 mL of distilled water. Malstat and nitroblue tetrazolium/phenazine ethosulfate (NBT/PES) mixture were used as substrate and colorimetric reagent in the assay system. The blood mixture was mixed with the substrate and colorimetric reagent. During the assay, changes of colour were monitored using a microplate reader at 655 nm (Fluostar Optima) after one-hour incubation. Based on the raw absorbance readings, a sigmoidal curve was plotted using GraphPad Prism 5 (GraphPad Software, Inc., San Diego, CA) to determine the 50% inhibitory concentrations (IC_50_) of the tested compound. Experiments were performed in triplicate and repeated three times.

### 2.2 *In vitro* cytotoxic test and selectivity indexes calculation

Vero (ATCC: CCL-81), normal epithelial monkey kidney cells were obtained from the American Type Culture Collection (ATCC), USA. Cytotoxicity of ar-turmerone was evaluated using the 3-(4, 5-dimethylthiazol-2-yl)-2, 5-diphenyltetrazolium bromide (MTT) assay in Vero cells seeded at 2 × 10^4^ cells/mL in DMEM complete medium (Gibco, Life Technologies, USA) [3]. The concentration of ar-turmerone and chloroquine diphosphate ranged from 0.1 to 10000 μM was used in the assay system. Untreated cell cultures were used as a control. The cell culture was incubated in the presence of test compounds for 48 h (37 °C, 5% CO_2_). MTT reagent was used as a colorimetric reagent in this assay and then the reagent was added to each well before 3 h incubation. The medium in the mixture was then removed and MTT formazan product was then dissolved in dimethyl sulphoxide (DMSO). Absorbance was measured at 540 nm (Fluorostar OPTIMA). The concentration of test compounds at 50% growth inhibition (IC_50_) was determined from a sigmoidal curve generated from a statistical analysis software, GraphPad Prism 5. Selectivity indexes were measured as the ratio of cytotoxic over antiplasmodial activity, each one expressed with IC_50_ (ratio IC_50_ MTT/IC_50_ pLDH assay) [4]. When a compound exhibits an SI value more than 10 (SI>10), the compound is generally considered as a promising antimalarial agent, non-toxic and safe which indicating that the pharmacological effect shown is not due to the cytotoxic effect [4]. Lower SI value suggests that the compound has low antiplasmodial activity and high toxicity.

### 2.3 Stage-specific susceptibility test

The stage-specific developmental inhibition of ar-turmerone was studied according to an established protocol [5]. Briefly, *P. falciparum* 3D7 was exposed to ar-turmerone at the concentration of IC_90_ (463.5 µM) for 5 h at each stage; ring stage (at 0 to 5 h), trophozoite stage (at 28 to 33 h), and schizont stage (at 42 to 47 h). Chloroquine diphosphate was used as a reference drug at the IC_90_ value (0.052 µM). The IC_90_ value used in the study was to evaluate the maximum parasite inhibitory effect of ar-turmerone and to compare the compound potency with the reference antimalarial drug, chloroquine diphosphate. After incubation, parasites were washed with incomplete medium (medium without Albumax I) to remove the test compound and were then further cultured using normal culture conditions (growth medium supplemented with Albumax I). Untreated synchronous cultures served as a control and were processed in the same way as the treated cultures. After the next ring cycle, blood smears were stained with Field A (methylene blue) and B (eosin) staining. Parasitaemia level in treated cultures was measured by counting the number of new rings formed in a total of 2 000 cells during the second intraerythrocytic cycle. The parasitaemia counting was carried out to observe the effect of the treatments given at the different stages (ring, trophozoite and schizont). Chemo-suppression percentage was calculated from the parasitaemia level of treated cultures as compared to a control. The stage counting was done blinded to treatment. Morphological changes were observed at each stage by microscopic examination of thin and thick blood smear of the treated and untreated cultures at 1000 × magnification using immersion oil.

## CRediT Author Statement

**Amatul Hamizah Ali:** Methodology, Writing–Original draft preparation. **Hani Kartini Agustar:** Writing, Reviewing, Editing. **Nurul Izzaty Hassan:** Revising. **Jalifah Latip:** Revising. **Noor Embi:** Supervision. **Hasidah Mohd Sidek:** Supervision.

## Declaration of Competing Interest

The authors declare that they have no known competing for financial interests or personal relationships exist.
